# What does qualitative evidence tell us about how having a diagnosis of type 1 diabetes mellitus impacts an individual’s identity? A systematic review

**DOI:** 10.1177/13591053251362032

**Published:** 2025-08-28

**Authors:** Rebecca Ades, Julie Evans, Jennifer Heath

**Affiliations:** 1 University of Hertfordshire, Hatfield, Hertfordshire, UK; 2 North London NHS Foundation Trust, UK

**Keywords:** identity, type 1 diabetes mellitus, T1DM, qualitative research

## Abstract

Despite literature acknowledging the significance of identity, thorough understanding of how Type 1 Diabetes Mellitus (T1DM) impacts identity, and individuals’ responses to this, is absent. This systematic literature review gathers, organises and evaluates existing qualitative studies investigating or reporting on how diagnosis of T1DM has impacted an individual’s identity, and the ongoing impact that diagnosis of T1DM has on an individual’s identity. Thematic synthesis of evidence from 13 studies produced four overarching themes: Social Identity; Loss of Self; Rejecting T1DM from Identity; and Acceptance into Identity. Findings highlight that T1DM impacts various aspects of an individual’s identity and individuals may respond to this in a range of ways, including struggling to adhere to T1DM management. It is of significant importance that healthcare teams expand the way they think about and work with T1DM, developing appropriate psychological interventions, including conversations about identity and a broader systemic focus on T1DM stigma.

## Introduction

Type 1 diabetes mellitus (T1DM) is a chronic illness characterised by destruction of beta-cells in the pancreas that produce insulin (Norris et al., 2020). Impaired insulin production and secretion leaves the body unable to control blood glucose independently, resulting in experiences of hypoglycaemia and hyperglycaemia (Atkinson et al., 2014). T1DM results in individuals having to monitor blood glucose via a capillary blood glucose monitor or continuous glucose monitor and manually administer insulin via injection (Holt et al., 2021). Worldwide, 5%–10% of cases of diabetes are type 1, translating to 21–42 million people (World Health Organisation (WHO), 2016); it is one of the most common chronic illnesses in children (Green et al., 2021).

The constant requirement for an individual to consider how food/beverages could impact their blood glucose, having to check blood glucose, administer insulin and worrying about the potential complications of the illness, places significant psychological and practical demands on an individual (Powers et al., 2017). A measure of how well one copes with and psychologically adjusts to these demands is the concept of illness integration (Felton and Revenson, 1984).

Illness integration refers to the extent to which an individual accepts and merges experiences of their illness into daily life, and with past and present identities and roles (Westra and Rodgers, 1991). Higher levels of illness integration have been positively correlated with treatment adherence, whereas low levels have been associated with avoidance of T1DM, low treatment adherence and greater diabetes complications (Commissariat et al., 2020; Oris et al., 2016).

Erikson (1959) proposed that a healthy identity is when an individual has integrated all parts of themselves into a coherent sense of self. To integrate T1DM into daily life, individuals need to integrate it into their sense of self and identity (Oris et al., 2016). Further, developing a strong sense of identity when one has T1DM has been associated with improved coping with the illness and protection against diabetes-related distress (Commissariat et al., 2020; Luyckx et al., 2008; Oris et al., 2016).

Chronic illnesses, including T1DM, can prevent the illness integration process as they can disrupt who a person thought they were. For example, the identity of individuals withT1DM has been found to be stigmatised due to its confusion with type 2 diabetes mellitus (T2DM), meaning those with T1DM are also falsely assumed to have caused their diabetes due to being uncontrolled around food and lazy (Liu et al., 2017). The stigmatised identity associated with T1DM may not align with the values and goals of the individual and therefore does not become integrated into identity (Charmaz, 1983; Oris et al., 2016). Furthermore, the ability to engage in tasks that explore and develop identity has been found to be significantly restricted by T1DM and its management (Luyckx et al., 2008; Sanders et al., 2019). For instance, socialising provides opportunities to explore and develop identity but can be hindered by T1DM as some individuals aim to conceal their diagnosis in public (Monaghan et al., 2015). The hindrance of T1DM on identity development may, however, be reducing with the advances of continuous glucose monitors (CGM) and young people showing greater positivity towards having and showing their CGMs (Beasant et al., 2023).

The encompassing nature of T1DM creates different reactions in individuals in terms of how they perceive T1DM and whether they are willing to integrate this into their identity. Oris et al. (2016) argued that individuals with T1DM can have one of four identity profiles: acceptance, enrichment, engulfment or rejection. Acceptance is when an individual accepts T1DM as part of their identity amongst other elements (Luyckx et al., 2008). Enrichment is where various positive experiences are taken from T1DM that have allowed the individual to enrich their identity, such as an increased appreciation for life and greater resilience (Tedeschi and Calhoun, 2004). Engulfment is when an individual perceives T1DM to dominate their identity (Morea et al., 2008), and rejection of T1DM from identity may result from an individual intensely disliking the illness and fearing others may only focus on their diabetes (Commissariat et al., 2023; Oris et al., 2016).

Accepting T1DM into identity, and/or viewing it as something that enriches identity improves treatment adherence and psychological wellbeing and reduces physical complications (Morea et al., 2008). Engulfment, and particularly rejection, has been associated with poorest treatment adherence and psychological well-being, and higher levels of diabetes related complications (Commissariat et al., 2023; Oris et al., 2016).

Despite academic literature acknowledging and exploring the significance of identity in the field of T1DM, thorough understanding of how individuals with T1DM perceive the impact diabetes has on their identity, and subsequently respond to this impact, is absent. Therefore, this systematic literature review aims to thematically synthesise research exploring or discussing the impact of T1DM on identity to gain a greater understanding of this area.

## Method

Due to this article detailing a review of literature, there are no human participants in this article and informed consent was not required. Systematic searches were conducted in March 2024 using CINAHL Plus and PubMed bibliographic databases, chosen as they incorporate literature from relevant disciplines including medicine, life sciences and applied social sciences. The SPIDER (Sample, Phenomenon of Interest, Design, Evaluation, Research Type) search strategy tool for qualitative research (Cooke et al., 2012) informed development of search terms to obtain as many relevant studies as possible. Supplemental Table 1 provides details of search terms used, with inclusion and exclusion criteria provided in Supplemental Table 2. Author RA screened all articles and extracted data based on Cochrane’s guidance for extraction of qualitative data (Noyes and Lewin, 2011). Data extracted included: authors, title, year of publication, aims/purpose of the study, methodology, participant demographics (diagnosis, gender, ethnicity, time since diagnosis, form of diabetes management) and key findings. The review included articles that reported, to any extent, how T1DM impacted identity of individuals with T1DM. This reporting could be in the form of participant experiences, feelings, views, ways of coping, adjustments, relationships with others, themselves or their T1DM or how they have/have not integrated T1DM into their identity. Studies that did not report any findings related to identity and T1DM were excluded.

### Search process

**Figure 1.**
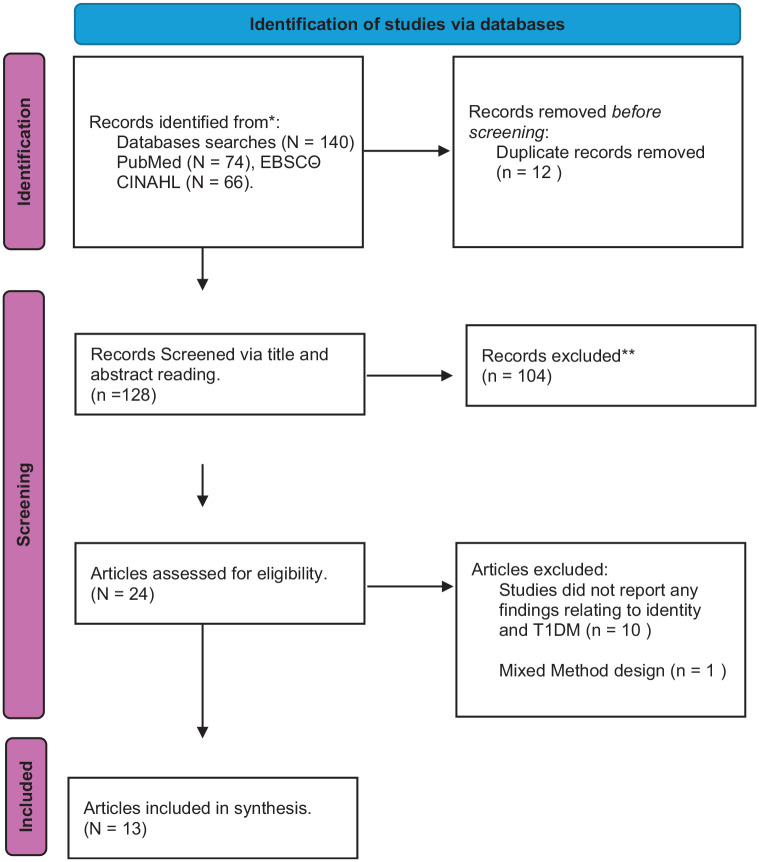
Search strategy. PRISMA diagram demonstrating the process of study selection. Source: Page et al. (2021).

Figure 1 presents a flow chart of search results and PRISMA screening process (Page et al., 2021).

#### Assessing study quality

Within this review, the GRADE-CERQual (CERQual: Lewin et al., 2018) was used to assess and describe the level of confidence that can be placed in each review finding by assessing four components: Methodological limitations, Coherence, Adequacy, and Relevance.

To appraise the quality of the 13 primary studies, and assess the methodological limitations aspect of the CERQual, this review used The Critical Appraisal Skills Programme (CASP) checklist for qualitative studies, as recommended by Munthe-Kaas et al. (2018). The quality of each study was assessed by RA, and this assessment contributed to the overall confidence rating given by the CERQual. Coherence, defined as the fit between the original data and the themes derived from this review’s findings (Colvin et al., 2018), was also assessed. This involved comparing original text segments that sub-themes and themes were inductively built upon, taking account of any contradictory data or alternative explanations. Assessment of adequacy required a review of the extent of detail in information articles provided to allow the reviewer to interpret the meaning and context of what was being researched, and the quantity of studies that made up a sub-theme (Glenton et al., 2018). Relevance of each study was assessed by reviewing the fit between the context of a primary study and the review’s research question, reviewing the population, setting and phenomena of interest for each study (Noyes and Lewin, 2011). Once rated, sub-themes were assigned an overall confidence level of either high, moderate, low or very low (Lewin et al., 2018). This approach to confidence rating assumes that each finding is of high confidence and should only be rated down if there are concerns in one or more of the CERQual components (Lewin et al., 2018).

#### Synthesis strategy

Thematic synthesis was used to synthesise the data collected from the 13 articles. This method has three stages: coding the findings of the primary studies, organising codes into descriptive themes and developing analytic themes (Thomas and Harden, 2008). Thematic synthesis allows systematic reviews to identify key themes and messages from a qualitative body of research, subsequently leading to greater understanding of an area (Nicholson et al., 2016; Seers, 2012).

## Results

The initial search identified 140 articles, 12 duplicates were removed. Screening titles and abstracts against inclusion criteria removed a further 104 articles, with 24 articles remaining for full-text screening. Of these, 13 met criteria for inclusion in the review. Table 1 details the authors, dates, country of the study, number of participants, their age and gender and which studies supported and provided confidence in the synthesised themes.

**Table 1.** Details of included studies (authors, dates, country of the study, number of participants, their age and gender) and support for thematic synthesis.

Study number	Study details				Themes Supported			
	Authors and year	Country	Number of participants	Participant age, gender, and years since diagnosis.	Social Identity	Loss of Self	Rejecting T1DM from Identity	Acceptance into Identity
1	Abdoli et al. (2013)	Iran	*N* = 26 overall *N* = 8 with T1DM *N* = 5 with family members of someone with T1D. *N* = 13 people without T1DM	Age range: 16–36 *N* = 7 Female *N* = 1 Male Range of years since diagnosis: 3–16 years	Supported	-	-	-
2	Abdoli et al. (2017)	USA	*N* = 9	Age range = 18–30 *N* = 6 Female *N* = 3 Male Years since diagnosis: not reported	Supported	Supported	Supported	Supported
3	Chalmers et al. (2022)	USA	*N* = 35	Age range:13–18 Mean Age: 14.9 years *N* = 17 Female *N* = 18 Male Years since diagnosis: not reported	Supported	Supported	-	-
4	Commissariat et al. (2016)	USA	*N* = 40	Age range: 13–20 Mean age: 16.5 ± 1.89 years *N* = 17 Female *N* = 23 Male Years since diagnosis: not reported	Supported	Supported	Supported	Supported
5	Dovey-Pearce et al. (2007)	UK	*N* = 19	Age range: 16–25 Mean age: 19.9 years *N* = 11 Female *N* = 8 Male Years since diagnosis: not reported	Supported	Supported	-	-
6	Fioretti and Mugnaini (2022)	Italy	*N* = 30	Age range: 18–24 *N* = 19 Female *N* = 11 Male Years since diagnosis: not reported	-	Supported	-	Supported
7	Goldman and Maclean (1998)	Canada	*N* = 30	Age range: 20–76 *N* = 17 Female *N* = 13 Male Range of years since diagnosis: 1–37	Supported	Supported	Supported	Supported
8	Kim (2022)	Korea	*N* = 12	Age range = 13–19 −16.7% aged 13–15 −50% aged 15–17 −33.3% aged 17–20 *N* = 7 Female *N* = 5 Male Years since diagnosis: not reported	Supported	Supported	Supported	Supported
9	Maietta (2021)	USA	*N* = 11	All over age 18 Mean age: 45.6 years *N* = 4 Female *N* = 7 Male Mean years since diagnosis: 28.7	Supported	Supported	Supported	Supported
10	Montali et al. (2022)	Italy	*N* = 22	Children and adolescents (aged 10–17) and young adults (aged 18–30) *N* = 15 Female *N* = 7 Male Mean years since diagnosis: 13	Supported	-	Supported	Supported
11	Sanders et al. (2019)	UK	*N* = 47	Age range: 16–24 Average years since diagnosis: 9	Supported	Supported	Supported	Supported
12	Tilden et al. (2005)	UK	*N* = 1	Aged 26 years Female Years since diagnosis: 16	-	Supported	Supported	-
13	Williams (1999)	UK	*N* = 20	Age range: 15–18 *N* = 10 Female *N* = 10 Male Years since diagnosis: not reported	-	Supported	Supported	Supported

### Summary of findings related to research quality

A wide range of topics were explored with participants from different contexts, but all articles encompassed how a diagnosis of T1DM impacts an individual’s identity, as well as the impact of societal stigma of T1DM on identity and diabetes-management behaviour. All studies were appropriately designed using appropriate methodology, and only one article (9) failed to explicitly report the study aim(s). To be as inclusive as possible, avoiding excluding studies that contribute valuable data to the synthesis, and because a lack of reporting does not always mean the research was poor quality (Aveyard, 2019), study 9 was included in the review. Another noteworthy point is that only two of the 13 articles considered and commented on the researcher’s relationship with the participant group and the data collected and analysed (6; 11). Commentary on reflexivity is important in qualitative research to acknowledge bias, as epistemology and positionality influence the research process and practice. Finally, all 13 studies made explicit statements of how their findings linked to their research objectives.

### Included study aims

Research aims of the studies encompassed exploration of experiences of how T1DM impacts identity or sense of self, how individuals may/may not integrate T1DM into their identity, how individuals feel towards or experience their T1DM or how societal stigma impacts identity. In addition, studies that explored diabetes management, treatment adherence, or diabetes-related self-care behaviours were included, as existing literature has highlighted struggles with identity/integrating T1DM into identity as contributing factors to low treatment adherence (Oris et al., 2016).

### Synthesis of findings

Prominent or recurring themes were constructed into patterns of meaning specific to the experiences of the impact of a diagnosis of T1DM on identity. Findings were synthesised into the following themes: Social Identity; Loss of Self; Rejecting T1DM from Identity; and Acceptance into Identity.

#### Theme 1: Stigmatising narratives and identity

T1DM impacted the social identity of participants. Stigmatised social narratives surrounding T1DM influenced negative attitudes held by others towards T1DM, which subsequently impacted behaviour towards those who live with it. Stigmatised narratives, attitudes, and behaviours of others were internalised by individuals, negatively shifting their perception of identity.

##### Stigma

The reviewed studies found substantial stigma surrounding T1DM that participants had been directly and indirectly exposed to. This stigma includes how the diagnosis was spoken about and how others responded to participants once they knew of their diabetes (2; 3; 7; 8; 11). Participants shared stigmatised, judgemental views and comments they had faced (2; 3; 8; 11), such as ‘*most people think you have to be overweight to have diabetes*’ (3), with one participant being told their T1DM was because they ‘*were overweight, or …did something bad to yourself or your parents didn’t take care of you*’ (2). These views were attributed to the ‘*stigma that’s attached itself to type* 1’ (11). Some participants reflected this stigma was due to public confusion between T1DM and T2DM (2), although this is further evidence of misunderstanding of the aetiology of distinct types of diabetes and perpetual stigma. Participants’ accounts reflect how the consequences of stigma mean they are met with blame and shame instead of empathy (2; 7; 8).

##### Attitudes and behaviours of others towards T1DM

There is consensus across the articles that participants with T1DM are treated differently by others *because* of their diabetes (11; 10; 2; 1; 8; 5; 9; 4; 6). Generally, participants experienced increased attention around their T1DM as negative; participants shared being viewed as ‘*different’, ‘sick’, ‘a patient who can do nothing’* (1), ‘*disabled’ and/or ‘less abled’* than others (1; 2; 4; 5; 8; 10; 11). One participant gave the example of being given ‘*semi-skimmed’* instead of ‘*full-cream’* making them ‘*…a reject because’* they were *‘different to everyone else’* (5).

For some, different treatment was in the form of well-intentioned gestures, such as posters about diabetes emergencies in places (e.g. classrooms) they frequented (5). In other cases, being treated differently led to alienation from social groups, bullying, and discrimination (1; 2; 4; 8; 10). For example, a participant shared, ‘*the human resources office called me and said, “we found out you have diabetes and we do not want sick people”’ (10)*. Negative views about a person having T1DM could be linked to Western, capitalist views that the value of an individual is measured by their contribution to society. Having T1DM could be viewed as a barrier to this contribution and thus be a factor in why participants of these studies have been viewed and treated as ‘*less able’* than those seen as healthy. A participant stated, ‘*to them you have a disability…“you can’t work like the rest of us”*’ (2). This interpretation is supported by nine of the 13 reviewed studies finding that participants concealed T1DM in public settings. Participants did this to avoid being socially grouped as ‘diabetic’ or viewed as ‘*weaker*’ and a ‘*liability*’, particularly in work environments. Instead, participants wished to appear ‘*normal and healthy*’ (1; 3; 4; 6; 8; 9; 10; 11; 13). One participant described concealing T1DM as helping them ‘*feel more like… I don’t like to use the word normal person… yeah not be like they’ve got something wrong with them’* (11). Such findings highlight the impact attitudes have on individuals, and negative experiences often influence behaviours regarding the management T1DM.

##### Internalised narratives

Some participants expressed viewing themselves as ‘*abnormal*’ and ‘*unwell*’ (4; 11; 8; 5), others believed that T1DM ‘*was my one flaw*’ (8). Participants expressed a drastic shift in how they viewed their identity after diagnosis of T1DM. A participant described feeling as though they were ‘*a very strong person*’ and suddenly became *‘a weakling’* (7) following diagnosis.

The sudden shift from viewing oneself as ‘*strong*’ to ‘*weak’*, alongside other participants viewing themselves as ‘*flawed*’ and ‘*abnormal*’ following diagnosis, links with social narratives recognised in sub-themes surrounding stigma and the attitudes of others. This finding indicates how societal stigma and attitudes of others towards T1DM and ‘*illness*’ can shape social identity, which could be internalised, shaping the way people with T1DM view themselves.

The process of internalisation could mean that a diagnosis of T1DM, and experiences of stigma surrounding it, might make someone feel ‘*different*’ from those around them. This ‘*difference*’ is reinforced by how others act and treat those with T1DM, which may make labels such as ‘*abnormal’* more salient, thus disturbing how someone views themselves and their identity.

#### Theme 2: Loss of previous identity

Participants articulated that T1DM disrupted their established identity. T1DM replaced their identity with a ‘*diabetic identity*’, thus creating a sense of loss towards their previous identity.

##### Disruption to identity

Participants expressed that, prior to their diagnosis they had established an identity, which T1DM disrupted because of a discrepancy between this identity and the one attached to a diagnosis of T1DM (4; 5; 7; 9; 8; 11; 12; 13).

Several factors contributed to identity disruption. Some participants had preconceived ideas about T1DM and what it means about a person, therefore the diagnosis disrupted their established idea of self. This is depicted when a participant shared that T1DM led them to think they were ‘*not who they thought they were*’ (7). Significant lifestyle changes resulting from constant monitoring and regulation of blood glucose, and the adverse physical consequences of mismanaging T1DM, can disrupt performance of daily tasks and therefore identities one wants to present in specific environments (e.g. at work [9]). For example, one participant expressed wanting to portray a ‘*student identity*’ during a presentation but experienced hypoglycaemia and instead felt ‘*unprofessional*’ (9).

##### Defined by a diabetic identity

Participants felt their identity was defined by T1DM (2; 3; 6; 7; 8; 10; 12). This was partly conveyed through participants regularly referring to themselves as a ‘*diabetic’* (7, 8) and was demonstrated through others identifying participants only by their diabetes (2; 3; 5; 6; 12). For example, a participant said their diabetes ‘*identified me a lot among my friends: I am the diabetic one… my identity is dictated by my condition’* (6). Similarly, another participant shared the *‘[hospital] said “oh you’re just diabetic”*…*I went into hospital a person and came out with this label*’ (5).

Participants shared that, to others, diabetes came above everything else in their life (6; 12). These participants subsequently expressed a sense of loss from previously being identified as a whole person, to only being identified by their diabetes. This was echoed where one participant previously identified themselves as a ‘*woman, a girl, mother*’ and lost those identities, now only thinking of themselves as ‘*diabetic*’ (7).

#### Theme 3: Rejection of T1DM

Some participants described a desire to reject T1DM from their identity. Many articulated the identity that they associated with T1DM violated their established identity, resulting in them rejecting T1DM via externalising it and not adhering to their diabetes management regimen.

##### Violating identity

For many, the identity attached to T1DM violated their preferred identity (7; 8; 9; 10; 12; 13). Participants expressed not wanting to accept the ‘*diabetic’* identity as they viewed it as ‘*giving up*’, fearing that if they did accept it ‘*it’s going to pull me down with it. I’ll feel tired and weak*’ (13), whilst others believed acceptance would ‘*banish*’ (8) them from themselves. Some believed their diabetes violated their identity in specific situations, such as at work, preventing them from being who they ‘*ought to*’ be (8; 9; 12; 13).

The violation T1DM has on participants’ identity, seems to also extend to future selves (6; 7). For example, a participant shared ‘[after being diagnosed] *I had grand plans for the future, and I could see them going down the toilet’* (7). This implies that the identity of T1DM is viewed to be an intolerable violation of what individuals would like their identity to be, which was therefore managed by actively rejecting it (8; 9; 12; 13). This view also appears to be dichotomous; one either accepts T1DM or continues to pursue the life one wants as, once diabetes is accepted, they cannot get their preferred identity back.

##### Externalising T1DM

Five articles evidenced how one way rejection of T1DM was facilitated was participants talking about and treating their T1DM as something external to themselves (2; 4; 7; 8; 13). Participants depersonalised their diabetes and spoke about ‘*it*’ as a separate part of their life, as opposed to something within themselves, for example *‘I don’t really check it as often’* (4; 7).

Permanently externalising T1DM could be a coping technique used to protect participants’ sense of self from the stigmatised identity of T1DM. It could also mean that T1DM is experienced as a foreign entity posing a threat to the self that individuals must continually work against. This idea is reflected in the wording used by some participants whereby they try to ‘*fight against*’ or ‘*run away*’ from T1DM, and to accept diabetes is to be ‘*defeated by it*’ (8; 13).

##### Poor management

Participants who were rejecting T1DM generally had poor management of their diabetes, which was defined as poor glycaemic control, increased episodes of hypo- and hyperglycaemia and increased diabetes complications (2; 4; 7; 12; 11; 13). Performing any behaviours associated with T1DM, particularly checking blood glucose and injecting insulin, were viewed as accepting T1DM into identity (7; 12; 13). Not performing diabetes self-care was also likened to wanting to ‘*rebel’* and ‘*not focus on’* management so they were able to ‘*focus on having fun*’ instead (2). Neglecting diabetes management, particularly in public settings, was therefore a method participants used to continually reject T1DM from their identity and focus on other things more consistent with their preferred identity.

Choosing to not adhere to diabetes management advice also seemed to be an act of rebellion against factors beyond diabetes itself. For some, rebellion was also against strict management routines and limitations imposed by healthcare professionals. A participant shared that their poor management was linked to the anger felt towards professionals who ‘*don’t give a sod about me… I’m just a number… I really want to feel like some sort of person*’ (12). Frustration was felt by participants when healthcare professionals incorrectly perceived non-adherence to be the result of poor choices and lack of knowledge of the dangerous side effects. Healthcare professionals reprimanded them and provided medical information they already knew, as opposed to exploring deeper causes of their non-adherence (11; 12). For others, the rebellion appeared to be against the life they viewed to be associated with the diabetic identity, which was one associated with limitations, a lack of socialising and being ‘*defective*’; instead, they wanted to pursue a life of socialising and fun (2; 4; 11).

#### Theme 4: Acceptance of T1DM into identity

Some participants shared their experiences of accepting T1DM into their identity. This predominately occurred through integrating diabetes and its management into their life. For some, acceptance was enhanced by seeing the positives of experiencing T1DM.

##### Integrating diabetes into identity

Participants argued that, to achieve integration of T1DM into their life they first had to recognise they had T1DM (2; 7; 11). Whether participants rejected or accepted T1DM into identity, they all recognised T1DM as a complex disorder that does not ‘*go away’* (8). For some, the permanence of T1DM resulted in acceptance as ‘*I know there’s nothing I can do except learn to take care of it*’ (4). This contrasts with those who rejected T1DM and viewed its permanence as a reason to reject it (8).

One participant speaks of this difference by sharing *‘…most people do not want to accept it. Some people give up. But I just do it… how can I not do it?’* (8). An important factor in integrating T1DM into identity was the acquisition and application of personal knowledge, including how to manage it and what it is like to live with, as opposed to simply accepting the views that others, such as healthcare professionals and parents/guardians, had imposed upon them (4; 11; 8). By developing personal knowledge and experience, participants were able to normalise T1DM, which helped them to integrate and accept it into their identity (10). When participants had acquired this knowledge and normalised view, viewing managing T1DM as aiding the pursuit of goals and performance, they were more accepting of T1DM as ‘*part of me now*’ (10), increasing integration and improving management (2; 4; 7; 13).

Acceptance and self-management of T1DM enabled participants to better meet values and goals linked with their preferred identity as their health was improved (2; 6; 7; 9; 10). Through these experiences, participants learnt that accepting and integrating T1DM into their identity did not result in their whole identity becoming about diabetes, instead diabetes became a background element to their life, leaving them able to do everything their ‘*healthy*’ peers could do (2; 4; 6; 7; 9; 10; 11;13). A participant shared that T1DM has not made them a ‘*new person’* but instead they stay the same but with *‘a new problem now’* (7). These findings are in stark contrast to those of the rejection theme where participants seemed to adopt more dichotomous views: choosing between managing their diabetes *or* doing all that they valued and enjoyed.

##### Positive aspects of T1DM

Participants who were judged to have accepted and integrated T1DM into their identity by the authors of the reviewed articles shared that T1DM had also added to their identity. T1DM made them recognise themselves as ‘*resilient’*, ‘*determined’*, ‘*empathetic’*, ‘*confident’*, and ‘*strong’*, which they did not feel prior to their diagnosis (2; 4; 6; 8). Having T1DM also made participants aware of the importance of their health and helped them to live a healthier lifestyle (2; 8). Additionally, participants shared that having T1DM made them feel more inclined to help others and, for one participant, helped to shape their future aspirations (2; 6).

Traits such as strength and resilience seem to directly oppose social narratives and stigma accompanying T1DM. The ability to take positives from experiencing T1DM, and apply them to identity, seems to require individuals to gather evidence against stigmatising views and to challenge them.

#### How the themes interact

**Figure 2.**
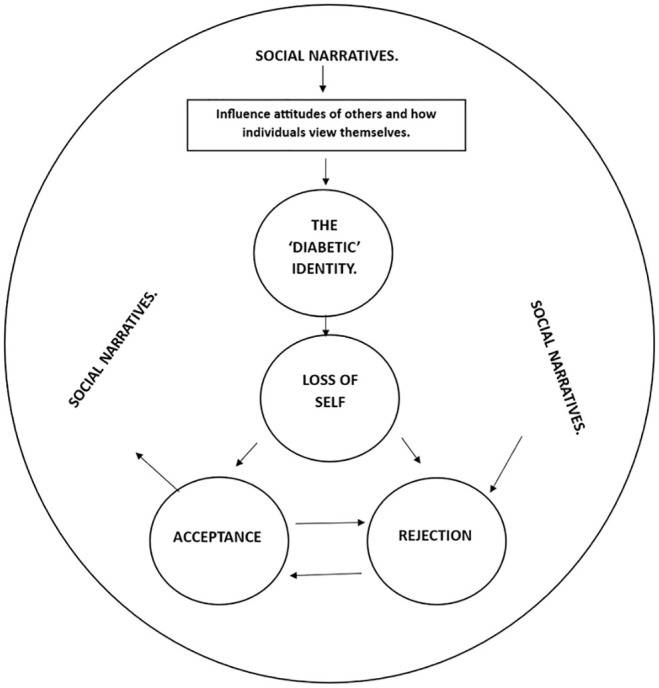
Diagram indicating *how the main themes of this literature review could interact*.

Wider social narratives can shape stigmatised views of T1DM, leading to preconceptions about how the condition affects a person’s traits and identity (see Figure 2) (1; 2; 4; 5; 6; 8; 10; 11). These assumptions influence how others perceive and interact with individuals diagnosed with T1DM and can result in internalised stigma (4; 5; 8; 11). The interaction between societal attitudes, self-perception and internalised stigma contributes to the formation of a ‘diabetic identity’.

This ‘diabetic identity’ often disrupts an individual’s pre-existing sense of self following diagnosis (4; 5; 7; 8; 9; 11–13). The perception that others focus primarily on their T1DM may cause people to feel that their identity is now solely defined by the condition, leading to a loss of self (2; 3; 6–8; 10; 12). This loss of self can then lead to two potential outcomes: rejection or acceptance of T1DM as part of their identity.

Rejection occurs when individuals are significantly affected by the stigma surrounding T1DM, which conflicts with their existing identity, prompting them to distance themselves from the condition (7–10; 12; 13). These individuals often have a dichotomous view of T1DM, believing that accepting and managing the condition means losing the parts of their identity they value most.

In contrast, acceptance happens when individuals acknowledge that while T1DM is a challenging, permanent condition, it doesn’t have to define their entire identity (2; 7; 11). A key factor in acceptance is gaining personal knowledge and experience in managing T1DM (4; 8; 11). As individuals become more adept at managing their condition, while continuing to live their lives, they begin to challenge the social narrative that they are ‘sick and unable’, allowing them to reclaim and maintain the parts of their identity that matter most.

## Discussion

This systematic literature review highlights the significant impact living with T1DM can have on an individual’s identity. The theme of ‘social identity’ supports previous findings that socialising can be difficult with T1DM due to people wanting to conceal their diagnosis (Monaghan et al., 2015). This theme can also offer explanation as to why individuals may wish to conceal their T1DM in public. T1DM has a stigma attached to it; the individual is blamed for their diagnosis (2; 3; 7; 8; 11). The stigma shapes how others think about and act towards those with T1DM, whilst also becoming internalised by the individual with T1DM (1; 2; 4; 5; 6; 8; 9; 10; 11). A wish to avoid socialising and/or conceal T1DM whilst in public can, therefore, be viewed as an attempt to avoid being assigned the social identity of T1DM.

The ‘loss of self’ theme supports previous ideas that living with T1DM can be disruptive to an individual’s identity (Charmaz, 1983; Oris et al., 2016). The theme also supports Oris et al.’s (2016) concept of ‘engulfment’ as an identity profile for T1DM; this review found that many individuals defined themselves only by their diabetes (4; 5; 7; 9; 8; 11; 12; 13). Findings highlighted that being defined by T1DM occurs not only on an individual level, but also through others only seemingly focusing on and caring about diabetes, which may encourage the view that T1DM is the most important aspect of identity.

Rejecting T1DM from identity supports Oris et al.’s (2016) identity profile of rejection. Rejection of T1DM also supports Charmaz (1983) and Oris et al. (2016) in their arguments that individuals may struggle to integrate T1DM into their identity when it violates their values (7; 8; 9; 10; 12; 13). Additionally, findings from this theme link the rejection of T1DM from identity with low treatment adherence, in line with previous findings (Commissariat et al., 2020; Oris et al., 2016). Findings from this review give greater insight into why individuals who reject T1DM from identity struggle with treatment adherence (2; 4; 7; 12; 11; 13). Poor treatment adherence was found to be a way to facilitate the rejection of diabetes; adhering to treatment was viewed as having taken on the diabetic identity, which violated values and the identity individuals wanted to have (7; 12; 13). It was also viewed as a form of rejection/rebellion against strict management routines and healthcare professionals, and the life associated with T1DM (2; 4; 11). In addition, this review found that individuals with T1DM felt frustration towards healthcare professionals when their poor adherence was viewed as simply poor decision making; they felt that services were not taking time to explore reasons behind non-adherence, such as struggles with identity as a result of living with T1DM, (11; 12).

The theme of acceptance supports Oris et al.’s (2016) identity profiles of acceptance and enrichment. It also supports the notion that accepting and integrating T1DM into identity is linked to better treatment adherence (2; 4; 7; 13). Findings from this review contribute to understanding of ways that acceptance is achieved. Individuals building their own knowledge-base and experiences of managing T1DM seemingly replaces views imposed upon them by others, which in turn normalises having T1DM (4; 7; 8; 11). For participants in the reviewed studies, accepting and managing diabetes ‘well’ did not lead to T1DM becoming their whole identity; instead, it facilitated them being able to follow their values and goals, which further facilitated acceptance (2; 4; 6; 7; 9; 10; 11; 12).

### Clinical implications

In considering what the available qualitative evidence communicates about how having a diagnosis of T1DM impacts an individual’s identity, this review has highlighted important implications for clinical practice when working with individuals with T1DM. It appears that many diabetes services focus mainly on the medical elements of T1DM (11; 12). These experiences can lead to individuals feeling as though their diabetes is the only part of themselves that is cared about (3; 12). In addition, strict management regimens and services reprimanding patients for not attaining high expectations set for adherence, due to assumptions that non-adherence is purely through patient-choice and a lack of understanding of the medical consequences of not managing it (11; 12), leads to some people feeling misunderstood by services and restricted by diabetes in terms of what they can do and eat; this results in a desire to rebel against medical professionals and the condition (3; 5).

The current approach of services to patient care takes a reductionist view that T1DM only impacts the body of the individual, whereas the findings of this review highlight how T1DM impacts individuals’ mental health, how they view themselves, how they behave and how others view and treat them. Therefore, services need to take time in appointments to speak with patients about how they are coping beyond the medical outcomes related to diabetes management/treatment adherence. Services need to recognise and appreciate the multiple complex reasons why individuals may struggle to adhere to expected diabetes management regimens and explore and work to address these with individuals.

Clinical psychologists have an important role in diabetes services and form part of the multi-disciplinary care offered to UK-based patients (NICE, 2022). The findings from this review imply the value of clinical psychologists within diabetes care exploring identity in assessment and formulation. If relevant for the individual with T1DM, identity may be a focus of psychological intervention.

The use of third-wave psychological therapies, such as Acceptance and Commitment Therapy (ACT) and Compassion Focused Therapy (CFT), may be useful interventions in helping those with T1DM who are struggling with identity. ACT could be used with individuals who believe having T1DM means they are unable to follow their values and goals in life, supporting them to incorporate activities into daily life that adhere to these values (Bennett and Oliver, 2019), expanding identity beyond ‘just’ T1DM. Techniques from CFT, such as building a compassionate self (Gilbert, 2009), may also be useful for individuals who may be experiencing significant amounts of shame and internalised stigma as a result of T1DM.

This review highlights that the impact of T1DM on identity extends beyond the individual with wider societal factors, such as stigma and attitudes of others, playing a significant role in the construction of the diabetic identity and how individuals subsequently respond to this. Therefore, it is important that services acknowledge and begin to consider ways to address these wider factors. For example, public health education, disseminating accurate information to address misconceptions and stigma, could be coproduced with diabetes service users.

### Strengths and limitations

This systematic literature review included primary studies that took place in various countries, recruiting even numbers of male and female participants. Participant demographics spanned a wide age range of 10–76 years, increasing transferability of results to the wider population of people with T1DM. In addition, despite this heterogeneity, the results of the studies consistently demonstrate how T1DM impacts identity, how participants respond to this impact, and highlight some of the influencing factors, indicating a robustness in the findings and the importance of considering identity in T1DM care.

Use of the CERQual tool showed that all of the sub-themes under the main themes were rated as having a high confidence level, with the exception of two with a moderate confidence level (externalising T1DM and being defined by a diabetic identity). The high confidence level was due to most of the studies that contributed to the sub-themes being appraised as having no concerns. For both sub-themes with a moderate confidence level, concerns came from authors not reporting or describing data that explicitly shared experiences of participants externalising their T1DM or feeling defined by it, meaning there were questions about the adequacy and coherence of the primary studies in relation to these two themes. Nevertheless, support for these sub-themes was found in studies without any concerns in the CERQual criteria, and the data reported by all primary studies noting these sub-themes was given in such detail that it allowed for interpretation and development of them.

Given the qualitative analysis conducted, another researcher using a different approach may identify different themes. Another limitation of the review may be that only two databases were searched. However, inclusion of both medical and life science journals better captured relevant research as opposed to databases, such as PsycInfo, which only captured social science journals and yielded few results (all of which were captured in the searches of PubMed and CINAHL). No date limits were set during the literature search meaning that four of the 13 included studies were over 10 years old. Experiences collated in that research may not represent social narratives and service experiences that are held today, such as young people’s experiences of T1DM being more positive due to the introduction of CGM (Beasant et al. (2023). In addition, the article screening process only involved author RA, which does not meet the Cochrane recommendations (Cumpston and Flemyng, 2024) increasing risk of bias.

### Implications for future research

This literature review focused on how T1DM impacts identity but it did not report on the consequences of this impact on individuals with T1DM. For example, individuals who reject their T1DM from identity have been found to have higher levels of mental health difficulties, such as depression and anxiety, both of which subsequently further impact participants’ relationship with their T1DM (Oris et al., 2016; Rassart et al., 2021). Further investigation into the consequences of impacted identity may provide a deeper understanding of the significance of identity in the field of T1DM. This is likely to highlight that the physical health of individuals with T1DM is not separate from their mental health, and it would be beneficial for services not to treat them as such.

### Conclusions

The findings of this systematic literature review convey the significant, complex, and dynamic impact T1DM has on an individual’s identity. This impact underscores the need for healthcare professionals to expand their thinking of T1DM, beyond being ‘just’ a medical condition, to one that that is shaped by wider social narratives and stigma and subsequently impacts nearly every aspect of an individual’s sense of self. This not only calls for healthcare services to be funded to have more time with patients to explore topics such as identity, but also the use of psychological practitioners within multi-disciplinary teams and public health campaigns about T1DM to begin to shift the stigmatising narratives surrounding the diagnosis.

## Supplemental Material

sj-docx-1-hpq-10.1177_13591053251362032 – Supplemental material for What does qualitative evidence tell us about how having a diagnosis of type 1 diabetes mellitus impacts an individual’s identity? A systematic review Supplemental material, sj-docx-1-hpq-10.1177_13591053251362032 for What does qualitative evidence tell us about how having a diagnosis of type 1 diabetes mellitus impacts an individual’s identity? A systematic review by Rebecca Ades, Julie Evans and Jennifer Heath in Journal of Health Psychology
