# Supplementary material for: What does qualitative evidence tell us about how having a diagnosis of type 1 diabetes mellitus impacts an individual’s identity? A systematic review
Source: J Health Psychol. 2025 Aug 28;31(3):983–1000. doi: 10.1177/13591053251362032 (PMC12949741; doi:10.1177/13591053251362032)
Supplement: sj-docx-1-hpq-10.1177_13591053251362032 – Supplemental material for What does qualitative evidence tell us about how having a diagnosis of type 1 diabetes mellitus impacts an individual’s identity? A systematic review [file sj-docx-1-hpq-10.1177_13591053251362032.docx]

**Supplementary Materials**

**Table 1:** *Full list of search terms used in accordance with the SPIDER diagram.*

| Sample: | type 1 diabetes OR type 1 diabetes mellitus |
| --- | --- |
| Phenomenon of interest: | identity OR illness identity OR self-concept |
| Design: | interview* OR focus group OR case study |
| Evaluation: | view OR experience OR feel OR coping OR psycholog* or adjustment or integration or relationship |
| Research: | Qualitative |
| Full list of terms. | **(type 1 diabetes OR type 1 diabetes mellitus) AND (identity OR illness identity OR self concept*) AND (view OR experience OR feel OR coping OR psycholog* OR adjustment OR integration OR relationship*) AND (interview* OR focus group OR case study) AND (qualitative)** |

**Table 2:** *Inclusion and Exclusion Criteria*

|  | Inclusion Criteria | Exclusion Criteria |
| --- | --- | --- |
| Sample | - Any individual with T1DM. - No age specification. - No gender specification. - No ethnicity specification. - No treatment type specification. | - Individuals with a chronic condition that is not T1DM. - Individuals with T2DM. - Family members, carers or peers of an individual with T1DM. |
| Phenomena of interest | Studies that explore:   - how T1DM impacts an individual’s identity or sense of self. - how individuals with T1DM may, or may not, integrate their diagnosis into their identity. - how individuals feel towards or experience their T1DM. - the impact of societal stigma of T1DM on identity or sense of self. - participant experiences or views of treatment adherence, diabetes management and/or diabetes related self-care behaviours. | - Studies that do not explore, in any form, the impact T1DM has on identity or sense of self. |
| Design | - Interviews - Focus groups - Case studies | - Questionnaire (including therapeutic outcome measures). - Survey. - Clinical trials. |
| Evaluation | Studies that report on:   - how individuals view, experience or feel towards their T1DM in relation to their identity. - how individuals have coped (or not) with having T1DM as part of their identity. - how individuals have (not) adjusted their identities based on their T1DM. - how individuals have, or have not, integrated their T1DM into their identity. - how a diagnosis of T1DM impacts psychological wellbeing in relation to identity. - how T1DM impact individual’s relationship with the self or others, in relation to identity. - the role of wider social stigma in how T1DM impacts identity. - identity in relation to treatment adherence, diabetes management and/or diabetes self-care. | - Studies that do not report any findings relating to identity. |
| Research | - Qualitative. | - Quantitative - Mixed method |
